# Evaluation of Root Dilaceration by Cone Beam Computed Tomography in Iranian South Subpopulation: Permanent Molars

**DOI:** 10.30476/DENTJODS.2021.91035.1547

**Published:** 2022-09

**Authors:** Bahar Asheghi, Safoora Sahebi, Maryam Zangooei Booshehri, Farnaz Sheybanifard

**Affiliations:** 1 Dept. of Endodontics, School of Dentistry, Shiraz University of Medical Sciences, Shiraz, Iran; 2 Dept. of Oral and Maxillofacial Radiology, School of Dentistry, Shahid Sadoughi University of Medical Sciences, Yazd, Iran; 3 Student Research Committee, School of Dentistry, Shiraz University of Medical Sciences, Shiraz, Iran

**Keywords:** Cone-beam computed tomography (CBCT), Dilacerations, Molar teeth

## Abstract

**Statement of the Problem:**

Root dilaceration is a developmental anomaly, which was first described in the 19th century as an abrupt change in the axial inclination between the crown and root of a tooth. Such an anomaly would potentially alter the dental therapeutic procedures and often demands special care. Hence, it is of great importance to diagnose dilaceration before starting any treatment procedure on the tooth.

**Purpose:**

The aim of the current study was to evaluate the prevalence of dilaceration in the first and second maxillary and mandibular molar teeth in Shiraz, Iran, using cone-beam computed tomography (CBCT) radiography.

**Materials and Method:**

In this retrospective study, 472 teeth from 141 CBCT images were studied. Each tooth was assessed by CBCT to diagnose dilaceration, its severity, direction, and anomaly location.

**Results:**

In this study, among 472 studied teeth, 136 teeth (28.8%) were diagnosed with root dilaceration. The most frequently affected tooth was the maxillary second molar tooth (45%). The results showed a statistically significant relationship between the gender of the patients and the prevalence of dilaceration. Most dilacerations were observed in distal direction, with a mild intensity, and located in the apical third of the roots.

**Conclusion:**

In this study, CBCT was presented as an advanced method for diagnosing dilaceration in maxillary second molar teeth and especially in the mesial root, which can be influenced by gender and it can play an important role in designing the treatment plans

## Introduction

First introduced by Tomes [ 1 ] in 1848, dilaceration is an evolutionary disorder between the mineralized and non-mineralized parts of the structure of a developing tooth. This anomaly appears as a deviation of the axial inclination of the crown and roots of a tooth and can occur anywhere along the length of the tooth from the crown to the root apex [ 2 - 3 ].

Considering the inevitable presence of some degree of curvature in most tooth roots, some studies have defined dilaceration as a certain degree of curvature from the normal dental longitudinal axis. For instance, Hamasha *et al* [ 4 ] and Malcic *et al* [ 5 ] considered dilaceration as the curvature of 90 degrees or more, whereas Chohayeb *et al* [ 6 ] regarded the curvature of 20 degrees or more as dilaceration. 

The development of dilaceration is associated with multiple parameters [ 7 ]. In this regard, it has been shown that the displacement of the root sheath of teeth following the gradient of bone remodeling within the alveolar bone can have a potential impact on the development of dilacerations [ 3 ]. Moreover, ethnicity and gender are crucial parameters to consider. Therefore, the prevalence of dilaceration has been studied in multiple ethnic groups. It has had different frequencies ranging from 0.3% to 98% in the studied teeth [ 4 - 6 , 8 - 9 ]. Another influential parameter in the development of dilaceration is trauma. It is worth noting that since dilaceration is very common in posterior teeth and trauma rarely affects this area, trauma was excluded from the list of influential parameters [ 4 , 10 ]. Two other determinants of dilaceration in posterior teeth are the genetic alteration of the tooth germ position and the effect of delayed eruption of teeth [ 5 , 9 , 11 - 12 ]. Nevertheless, they do not seem to be the only reasons for dilaceration in these regions [ 3 ].

With respect to the location of dilaceration, some studies declare that dilaceration is more common in the posterior regions of the mandibular [ 4 ] and permanent teeth [ 13 ], while others state that the maxilla is more involved [ 5 ]. It has been shown that dilacerations in incisors, canines, and premolars is more common in the apical third of the roots. In molars, dilaceration usually occurs in the middle third of the roots; while in third molars, dilaceration occurs more frequently in the coronal third of the roots [ 5 ]. Nevertheless, there is no consensus among researchers on the teeth, which are the most, and the least affected with this anomaly [ 14 ]. 

Considering the above-mentioned parameters, it is of great importance to diagnose dilaceration at the roots. Radiography is the most efficient method for this purpose [ 4 ]. Most previous studies have used conventional radiographies such as periapical and panoramic to investigate the prevalence of dilacerations [ 15 - 18 ]. However, these methods can take the images of the teeth only in two dimensions.

An appropriate method to overcome this shortcoming is to use cone-beam computed tomography (CBCT) imaging. CBCT imaging is a technique that has been widely used in various studies of root and canal system morphology to show the third dimension of the teeth [ 19 - 20 ]. In this imaging technique, all the distortions and superimpositions are removed, making it more appealing in various teeth anomaly studies [ 21 ]. Therefore, using CBCT radiography leads to an accurate identification of the location, direction, and degree of dilacerations [ 21 - 22 ]. Nevertheless, only a few studies (case reports) have exclusively used CBCT imaging to investigate the prevalence of dilacerations [ 23 - 24 ]. The aim of the current study was to use CBCT to investigate the prevalence of dilaceration in the teeth and roots of the first and second molars in the maxilla and mandible. Moreover, the severity, degree, and location of dilaceration in these teeth were studied. Furthermore, the relationship between the gender and the prevalence of dilaceration among patients referring to dental clinics in Shiraz, Iran, were investigated. 

## Materials and Method

### Sample collection

In this cross-sectional and retrospective study, the prevalence of dilaceration was assessed in randomly selected high-quality CBCT radiographic images archived from 141 randomly selected patients (18 to 65 years old). These data were gathered from the archives of four oral and maxillofacial radiology clinics in Shiraz from July 2017 to August 2020. The CBCT images were taken from patients with different conditions such as dental implantation, maxillary and mandibular bone fracture, or tumor. Therefore, additional radiation was avoided.

The current study was approved by the Ethics Committee of Shiraz University of Medical Sciences (IR.SUMS.DENTAL.REC.1398.33).

In this study, the presence of dilaceration in 141 CBCT images, 472 maxillary and mandibular molars, and 1204 molar roots was evaluated. Of all molars, 119 were maxillary first molars, 140 were maxillary second molars, 95 were mandibular first molars, and 118 were mandibular second molars.

In the present study, high-resolution CBCT images of the first and second mandibular and maxillary molars were selected. All images were taken from completely erupted teeth with closed and fully developed apexes. 

Furthermore, the supernumerary teeth, teeth with internal and external resorption, and deciduous or permanent teeth with open apex roots were excluded from the study. 

The data about the number of tooth with dilacera-tion, the patients’ gender, the position of the teeth with dilaceration in the jaw (maxilla or mandible), the pres-ence or absence of dilaceration in each root (categorized based on the type of root), the severity of dilaceration based on Santana’s classification[ 25 ]the direction of dilaceration (mesial, distal, buccal, palatal/lingual), the location of dilaceration (apical, middle, coronal), the teeth with S-shaped roots, and the presence of supernu-merary roots in the molar teeth and dilaceration in them were collected for statistical analysis. ], the direction of dilaceration (mesial, distal, buccal, palatal/lingual), the location of dilaceration (apical, middle, coronal), the teeth with S-shaped roots, and the presence of supernumerary roots in the molar teeth and dilaceration in them were collected for statistical analysis.

In the present study, the definition of dilaceration presented by Chohayeb *et al* [ 6 ] was used. They consider the deviations of more than 20 degrees from the longitudinal axis of the tooth as dilaceration. Moreover, according to the study of Santana [ 25 ], the intensity of the curvature was categorized as mild (with 20-40 degrees of deviation), moderate (with 41-60 degrees of deviation), and severe (with more than 60 degrees of deviation).

The bending angle of dilaceration was investigated using the method presented by Schneider [ 26 ]. In this approach, the angle between two hypothetical lines connecting the apex and the orifice to the beginning of the curvature is measured. 

In the current study, the presence of at least one root with dilaceration in the multi-rooted teeth was considered enough to mark that tooth as dilacerated. Furthermore, in teeth with multiple dilacerations in different planes, the largest angle was considered as dilaceration. 

All CBCT images were prepared using a Planmeca Promax 3D Mid device (Helsinki, Finland) at 90 kVp and 14 Ma, with an exposure time of 15 s, and automatically adjusted based on the patients’ body weight and size. A maximum field of view of 10*10 cm, a voxel size of 150 μ, and a high definition mode were used.

**Figure 1:**
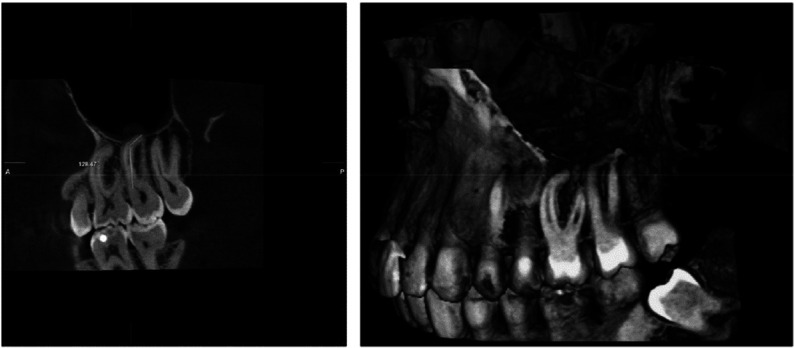
Moderate dilaceration in the mesial root of maxillary second molar in the sagittal (left) and 3D (right) planes

Root dilaceration was evaluated using a magnification tool in the Romexis software. The CBCT images in the axial, coronal, and sagittal sections were analyzed using the Romexis imaging software (version: 3.8.2) on a 32-inch monitor in dim light (Figure 1).

All CBCT images were inspected retrospectively by a calibrated endodontist and a senior dental student who were trained to interpret the images independently. There was a two-week interval between the assessments. Prior to the experiments, the investigators evaluated 60 other CBCT images. If any disparity was seen between the opinions, a radiologist evaluated the images in order to reach a consensus. To assess the intra-examiner reliability, a re-assessment was performed one month after the first session.

### Data analysis

The data in the current study were analyzed by the SPSS software (version: 18.0, SPSS Inc., Chicago IL, USA) and the figures were generated using the Graph-Pad Prism software (version: 8.0). The Chi-square statistic test and Fisher’s exact test were used to evaluate the qualitative data and the significance level was set at *p*<0.05. In this study, the p value was used to investigate whether root dilaceration was statistically dependent on gender, jaw type, tooth type, and root type. The prevalence of dilaceration, S-shaped roots, and extra roots in the studied teeth was presented as percentages. The agreements between the inter- and intra-examiners were calculated using Cohen’s kappa coefficient.

## Results

**Figure 2:**
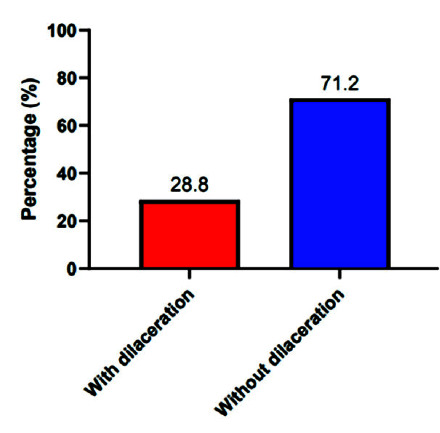
The prevalence of dilaceration in the studied teeth

It was observed that the overall prevalence of dilaceration in at least one root in the studied population was 51.7% (n=73). The prevalence of dilaceration in the first and second molars was 28.8% (n=136) (Figure 2). The assessments of the relationship between the prevalence of dilaceration and the patients’ gender revealed that dilaceration was statistically dependent on gender and occurred more often in females (*p*= 0.044) ( Table 1).

**Table 1:** The distribution of dilaceration between the two genders

	Female	Male	Total	*p* Value
Teeth without dilaceration	186 (67.6%)	150 (76.1%)	336 (71.2%)	*p* = 0.044
Teeth with dilaceration	89 (32.4%)	47 (23.9%)	136 (28.8%)	
Total	275 (100%)	197 (100%)	472 (100%)	

The results obtained in this study demonstrated that the prevalence of dilaceration was more in the upper jaw than in the lower jaw (*p*< 0.001) ( Table 2). The teeth most affected with this anomaly were the maxillary second molars (*p*< 0.001).

**Figure 3:**
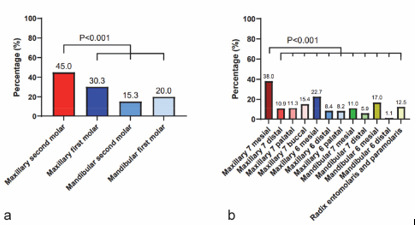
**a:** The prevalence of dilaceration in each studied tooth, **b:** Based on the root positions

The maxillary first molars, the mandibular first mollars, and the mandibular second molars respectively occupied the next ranks (Figure 3a). 

**Table 2:** The prevalence and distribution of the dilacerated teeth based on the jaws

	Maxilla	Mandible	Total	*p* Value
Teeth without dilaceration	160 (61.8%)	176 (82.6%)	336 (71.2%)	*p* < 0.001
Teeth with dilaceration	99 (38.2%)	37 (17.4%)	136 (28.8%)	
Total	275 (100%)	213 (100%)	472 (100%)	

**Figure 4:**
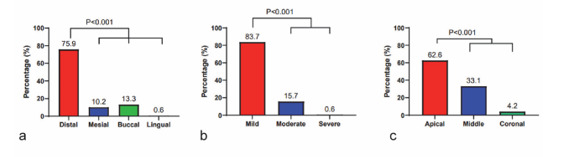
**a:** The direction, **b:** Severity, **c:** and prevalence of dilaceration in the root length

Investigating the roots of the above-mentioned teeth showed that dilaceration was more common in the mesial roots of maxillary second molars (*p*< 0.001) (Figure 3b). In general, it was shown that curvature and root dilaceration were more common in the distal direction mostly in the third apical with a mild intensity (*p*< 0.001) (Figure 4).

Out of 472 teeth, 2.1% had at most two extra roots in which the prevalence of dilaceration was 12.5% (n=1). Moreover, it was observed that 5.72% of all the teeth had S-shaped roots.

Regarding the inter-examiner’s agreement (after the training session), Cohen’s kappa coefficients for the first and second assessments were 0.991 and 0.993, respectively. The overall Cohen’s kappa coefficient for the intra-examiner’s agreement was 0.996. Taken together, this result suggests a very good agreement between the intra- and inter-examiners.

## Discussion

Dilaceration is recognized as one of the most important anomalies that have the potential to affect the success rate of endodontic treatments [ 4 , 6 , 27 ]. Frequent errors such as ledge, transport, zipping, and broken file are the results of abnormal root canal curvature, which in turn can affect the outcome of endodontic treatment procedures especially in molar teeth [ 28 ]. Hence, it is of great importance to diagnose dilaceration before starting any treatment procedure on the tooth. In severe cases of dilaceration, alternative endodontic treatment methods such as vital pulp therapy should be considered [ 4 , 6 ].

CBCT has been introduced in the current study as a novel, non-invasive, and accurate radiographic technique for the diagnosis of dilaceration. It enables dentists to examine the tooth in three dimensions. This method has some advantages over invasive techniques such as tooth extraction by providing the exact angle of dilaceration at the root. Moreover, CBCT imaging provides some information about the patient’s age, gender, and teeth symmetry [ 29 - 31 ].

In the current study, the different planes of the roots of the first and second molar teeth were investigated using CBCT images. Such planes are not visible in conventional radiographs. Here, the criteria presented by Chohayeb [ 6 ] and the classification of Santana *et al* [ 25 ] were used to define dilaceration based on root curvature. These criteria (deviation of >20) were also used in different thirds of the roots.

The literature review shows that there is a large discrepancy in the prevalence of dilaceration in different populations. It has previously been shown that dilaceration is the most common dental anomaly in the population of Iran, comprising 15% of all types of dental anomalies [ 32 ]. Due to the vastness of the country, few studies have investigated the prevalence of dilaceration in Iran and most of them have been conducted without CBCT radiography [ 17 - 18 , 32 ]. 

In the current study, the prevalence of dilaceration was reported among patients diagnosed with at least one case of this anomaly (51.7%). The teeth that had at least one root with dilaceration (28.8%) were also investigated.

Multiple studies assessed the prevalence of dilaceration in molar teeth using periapical or panoramic radiographies. They showed that the prevalence of dilaceration was below 9% [ 5 , 9 , 14 - 15 , 18 ]. A higher prevalence of dilaceration in molar teeth was observed in the present study (28.8%) than in previous studies. This may be due to the better diagnosis of buccal and/or lingual dimensions via CBCT imaging. Moreover, different sample sizes, different races, and different definitions of dilaceration were used in the current study, potentially affecting the final results.

In line with some previous studies [ 9 , 15 , 33 ], the current study showed that the presence of dilaceration was significantly dependent on the patients’ gender and was more common in females. However, some other papers showed that dilaceration was more common in males [ 32 , 34 ] or was even gender-independent [ 14 , 16 , 35 ]. It was observed that most teeth affected by dilaceration were the maxillary second molars. Irrespective of the third molars, a similar observation has been reported in two previous studies [ 16 , 36 ]. Meanwhile, excluding the third molars, some studies have shown a different result and have introduced other teeth such as maxillary first molars [ 5 , 14 ] or mandibular second molars [ 9 , 15 , 18 ] as the candidates with a higher prevalence of dilaceration. There are controversies among different studies regarding the location of dilaceration; however, two studies, which considered all teeth in their investigations, have mentioned the posterior region as the most common location of dilacerations [ 9 , 16 ]. 

In the current study, it was also observed that the prevalence of dilaceration was significantly higher in the maxilla than in the mandible as has been previously reported [ 5 , 9 , 36 ]. Although some studies have indicated that dilaceration mostly occurs in the mandible [ 4 , 15 ], previous investigations have not reported a difference between the two jaws [ 14 , 16 , 18 , 33 ]. 

Inspecting the roots separately showed that the highest prevalence of dilaceration was in the mesial root of the maxillary second molar. Nevertheless, the distal root of the mandibular second molar has also been introduced as a candidate with the greatest prevalence of dilacerations [ 18 ]. In the present study, the extra roots of mandibular molars as well as the buccal roots of maxillary second molars with two roots were also reported. Mandibular molar roots are referred to as entomolaris and paramolaris corresponding to teeth numbers 6 and 7, respectively [ 1 , 18 ]. The prevalence of dilaceration in the aforementioned roots is shown in Figure 3B. To the best of our knowledge, this was the first study to evaluate the prevalence of dilaceration in these teeth using CBCT radiography.

A dilaceration with a mild intensity was the most common dilaceration in the current study. This was consistent with the studies of Santana *et al* [ 25 ] and Erlich and Panella [ 37 ]. This was followed by moderate and severe intensities, respectively. Furthermore, examining the location of dilaceration on the roots of the teeth revealed that the highest prevalence was related to the third apical of the roots. This is in line with some previous studies [ 5 , 25 , 36 , 37 - 38 ] and in contrast with another research, which showed that dilaceration was more common in molars in the middle third [ 5 ].

The results of the present study confirmed those of previous studies and showed that dilaceration was mainly in the distal direction [ 25 , 34 , 39 ]. 

Due to the relatively high prevalence of dilaceration in this study and the vastness of Iran, these epidemiological data cannot be generalized to the whole country. Therefore, it is recommended that similar studies with larger sample sizes and in different parts of the country be conducted using CBCT radiography.

## Conclusion

Using an advanced imaging modality, the results showed that dilaceration was relatively common in the molar teeth and especially in the mesial root, which can be influenced by gender and the majority of participants suffered from some degree of curvature. Thus, it is highly recommended that dental practitioners evaluate the presence of dilaceration with the help of diagnostic imaging before starting any treatment on molar teeth. This is even more crucial when the patient is female or the teeth are maxillary second molars.

## Acknowledgement

The Ethics Committee of Shiraz University of Medical Sciences approved the current study (IR.SUMS. DENTAL.REC.1398.33). All methods were carried out in accordance with the institutional guidelines and regulations. The signed informed consents were obtained from the patients so that their radiographic images could be used in the current study.

## Conflict of Interests

The authors declare that they have no conflicts of interests.
